# Positive–Negative Asymmetry in the Evaluations of Political Candidates. The Role of Features of Similarity and Affect in Voter Behavior

**DOI:** 10.3389/fpsyg.2018.00213

**Published:** 2018-02-27

**Authors:** Andrzej Falkowski, Magdalena Jabłońska

**Affiliations:** Department of Psychology, SWPS University of Social Sciences and Humanities, Warsaw, Poland

**Keywords:** features of similarity, framing, reference point, negativity effect, similarity judgments

## Abstract

In this study we followed the extension of Tversky’s research about features of similarity with its application to open sets. Unlike the original closed-set model in which a feature was shifted between a common and a distinctive set, we investigated how addition of new features and deletion of existing features affected similarity judgments. The model was tested empirically in a political context and we analyzed how positive and negative changes in a candidate’s profile affect the similarity of the politician to his or her ideal and opposite counterpart. The results showed a positive–negative asymmetry in comparison judgments where enhancing negative features (distinctive for an ideal political candidate) had a greater effect on judgments than operations on positive (common) features. However, the effect was not observed for comparisons to a bad politician. Further analyses showed that in the case of a negative reference point, the relationship between similarity judgments and voting intention was mediated by the affective evaluation of the candidate.

## Introduction

The perception and interpretation of the world depends on the comparisons people make and reference points they adopt. There are no absolute evaluations – a judgment is always relative and context-based. Research on similarity judgments, preference formation and decision-making (Tversky and Kahneman, 1986) showed that the criteria on which people based their decisions were malleable and dependent on changing circumstances as well as the way a problem was formulated. The comparisons that people make – whether to a particular situation or to one’s expectations – were proven as the essence of psychological judgments regarding objects and experiences that take place in a competitive environment.

The point of reference created an interpretative frame for the evaluation of observed reality. As stated by Minsky (1975, p. 212), it constitutes “a remembered framework to be adapted to fit reality by changing details as necessary”. The classical theory of framing by Minsky laid the groundwork for research into cognitive processes such as perception, memory and conceptual thinking. Numerous studies about the effect of framing on evaluations and the effectiveness of persuasive messages in political and social contexts have shown different ways in which various frames – adopted by individuals or imposed by others – affect the perception of objects or events (i.e., Chong and Druckman, 2007; Cwalina and Falkowski, 2015).

In a political context, Bizer and Petty (2005); Bizer et al. (2011) investigated how differences in valence framing (positive or negative) affected the persuasiveness of political messages and voter behavior. The participants were asked to think either in terms of the support for a preferred candidate (positive valence framing) or their opposition toward the other (unwanted) politician (negative valence framing). The results indicated visible differences in the effect of positive and negative valence framing on evaluations of two candidates. Voters who were presented with an image of a negative candidate were much more certain of their decision than when they thought about their preferred politician. The observed certainty was a direct outcome of their attitude toward the preferred candidate modified by a positive or negative reference point that framed the choice context. Based on these results and the effect of framing on voting intention it may be extrapolated that the negative reference point drove voters further away from the unwanted alternative than the positive framing pulled them toward a preferred candidate.

Research conducted by Bizer et al. (2011) also gave insight into negative comparative advertising and its greater effectiveness compared to the persuasiveness of “positive” campaigns. The key to advertisement effectiveness lied not only in the unfavorable message about the attacked candidate but also in that the voter’s attitude toward the preferred politician was shaped by the negative image of his or her competitor. The effectiveness of such campaigns was explained by the simultaneous activation of these two forces that affected voter attitude toward the politician (the infamous one-two punch): not only was the image of the opposing candidate tarnished but there was also a rise in preference for the supported candidate. Such an effect was possible when the positive attitude toward one option was modified by the negative image of the unwanted alternative.

A similar mechanism was observed by Kaid (1997) in her studies on the effectiveness of negative political advertising in the 1996 presidential elections in the United States. Kaid selected a few political television commercials that presented a negative image of the opposing candidate along with visual distortions of the messages presented by him. Different groups of participants watched original negative advertisements of Dole and Clinton or the same spots but with the distortions removed. As expected, results showed that the unfavorable message had a negative influence on the attacked candidate and there was less willingness to vote for him. However, another effect noticed was that negative information also led to an increase in the preference for the candidate who used the negative ad. Moreover, this increase led to a higher resistance to change in attitude when voters evaluated their preferred candidate consistent with the result found by Bizer et al. (2011). Additional support for the findings is given by the well-known negativity effect summarized by Baumeister et al. (2001) in the title of their article, “Bad is stronger than good.”

The aim of this paper is to delineate and attempt to explain the psychological mechanism behind the negativity effect in voting behavior. As we have already mentioned, there are no absolute judgments because the evaluation is always performed in relation to some point of reference. Such a reference point can be positive or even ideal if one juxtaposes the candidate against one’s expectations in relation to the features of an ideal political candidate or negative if the comparison is made against the image of a bad politician depicted in a negative comparative advertisement. Taking that into consideration, we needed a theoretical framework that would explain such a comparison process.

The features of similarity or contrast model by Tversky (1977) seems to be an appropriate psychological theory for use in this case. The model assumes that the similarity between two sets of features was dependent on the number of common and distinctive features that made up the two sets. The greater number of common features and the lesser number of dissimilar features two sets contain rendered them more similar. But the similarity was not symmetrical and depended on the reference point. For instance, a political candidate A was more similar to the image of an ideal political candidate than the ideal politician was to the candidate in question. Alternatively, a candidate B with a less favorable profile was perceived as better if juxtaposed with the image of a bad politician. Hence, the similarity between two sets of features is a result of comparison between the actual candidate with an internalized image of an ideal or bad politician that served as the benchmark for evaluation. We propose that this theory can be used to explain the mechanism of the negativity effect.

Furthermore, the negativity effect can be explained with reference to a stronger emotional load of negative stimuli. Such an observation was made by Öhman et al. (2001) who in a series of experiments on stimulus perception showed that when presented together with neutral faces, an angry or anxious face was noticed faster than one displaying positive emotions such as joy. Negative facial expressions signalized a threat and they immediately attracted attention. Contrarily, stimuli that elicited positive emotions were not related to threat and were processed less attentively. Here again, the positive–negative asymmetry was clearly noticeable but in this instance, it pertained to emotional rather than cognitive processes. Nonetheless, in both cases the avoidance of a negative image seemed stronger than an approach toward a positive stimulus. Hence, the present study integrated the cognitive mechanism of the negativity effect delineated in the contrast model of similarity with its emotional component that resulted from the asymmetrical effect of positive and negative emotions on stimulus evaluation.

**FIGURE 1:**
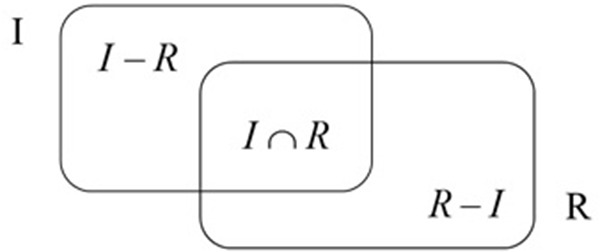
The relation between two sets of features (Tversky, 1977).

Tversky’s (1977) features of similarity was one of the most important contributions to the literature on similarity. According to the theory, stimuli are represented as collections of features and the similarity between them is described as a feature-matching process. The theory can be visually presented as two sets of features (**Figure 1**) where one of the sets (*I*) consists of ideal features which represent desirable characteristics of an object and the other set symbolizes a real object (*R*). The two sets overlap (*I* ∩ *R*) for features that are common for both sets.

The contrast model expresses the similarity between objects as a linear combination of the measures of their common and distinctive features that belong only to *I* and *R* (see **Figure 1**). Per the assumptions of the model, an increase in the number of common features leads to an increase in similarity and a decrease in difference. On the other hand, an increase in the number of distinctive features (that belong to one object but do not belong to the other) increases the difference and decreases the similarity.

The similarity can be expressed with the following equation (Tversky, 1977) that has been adjusted for the presented example:

s(I,R)=F(I∩R,I−R,R−I)

Similarity between the real object (*R*) and the ideal object (*I*) is described with a three-argument function: *I* ∩ *R*, features common for *I* and *R*; *I* - *R*, features belonging to *I*, not shared with *R*; and *R* - *I*, features belonging to *R*, not shared with *I*. The overlapping part common for the ideal object and the real object consists of positive features exclusively as the ideal object cannot incorporate any negative characteristics. Yet the distinctive features belonging to the real object (*R* - *I*) can be negative. An increase in the similarity of the real object to the ideal object can be achieved by extending the common part (I ∩ R) which implies adding positive features to the real object. On the other hand, a greater similarity may also be reached by diminishing the differences between the two objects by removal of negative features from the real object.

The original theory described the relationships between closed sets and was experimentally tested on artificial stimuli such as schematic faces, letters, or sequences of symbols. In these sets, with a defined number of features, the similarity of two sets *a* and *b* (defined by the number of common and distinctive features) is a linear function with the slope parameter -1, which assumes that the judgments of similarity and dissimilarity are complimentary. In this situation, an increase or decrease in similarity can be achieved by a manipulation of particular features so that they become common or distinctive.

However, in our physical and social reality, more often we encounter natural objects with characteristics that are more difficult to describe. Some features disappear entirely or are replaced by new ones and, over the time, they may be gradually weakened or enhanced. This point was raised by Falkowski et al. (2018) who extended the contrast model of similarity to open sets. As the name suggests, such sets have no boundaries and new features can be added and old ones removed (whereas in the original model the features were only shifted from the common set to distinctive and vice versa but could not be entirely deleted or added). For example, in the second half of the 19th century, European urban transportation services used horse and carriage which was fully abandoned and replaced by steam tramways and later electrical ones. Not until the 1950s was air conditioning entered into the set of features characteristic of public transportation. Different features of transportation have been strengthened or weakened in ways that brings it closer to the ideal image.

A similar situation can be observed in the political arena, where political candidates display different personality traits that more or less fit the image of an ideal political candidate. The strengthening of positive features such as competence and integrity would certainly bring a candidate closer to the image of a preferred politician, whereas strengthening undesirable traits such as lack of education or untruthfulness would distance him or her from the ideal. On the other hand, a reduction in such traits would have the opposite effect where decreasing the value of competence and integrity would distance the candidate further away from the image of an ideal politician and downplaying his or her lack of education or untruthfulness would bring the candidate closer to the ideal.

Consequently, it may be assumed that natural objects constitute open sets of features and the similarity between them results not from shifting the features between the sets, as it was in the case of closed sets, but from their activation or extinction. A political candidate may potentially have all personality traits that are present in the image of an ideal candidate with some of these features apparent, some less noticeable, and others fully latent (but if necessary, available for activation).

If the candidate wants to compare him/herself to the image of an ideal candidate, he or she can adopt one of the following strategies. In order to increase the similarity between the ideal and the real candidate, one can activate or strengthen positive features that the two sets share. Conversely, it is possible to weaken or decrease distinctive negative features that characterize the real candidate but not the ideal one. But if the negative image of the political candidate (the so called “anti-ideal”) serves as the reference point, the effective strategy would aim at maximizing the distance (dissimilarity) between the real politician and the reference point. This can be achieved either by the extinction (or reduction) of common negative features or the strengthening of distinctive positive features.

Within the contrast model of similarity framework, a simple simulation, in which common and distinctive features are either strengthened or weakened, can be conducted in order to present the effect of positive and negative features on judgments of similarity between the real political candidate and its ideal or negative image. Similarity between two sets should be calculated based on the ratio model which is a matching function describing the features of similarity (Tversky, 1977) depicted on **Figure 1**.

**FIGURE 2:**
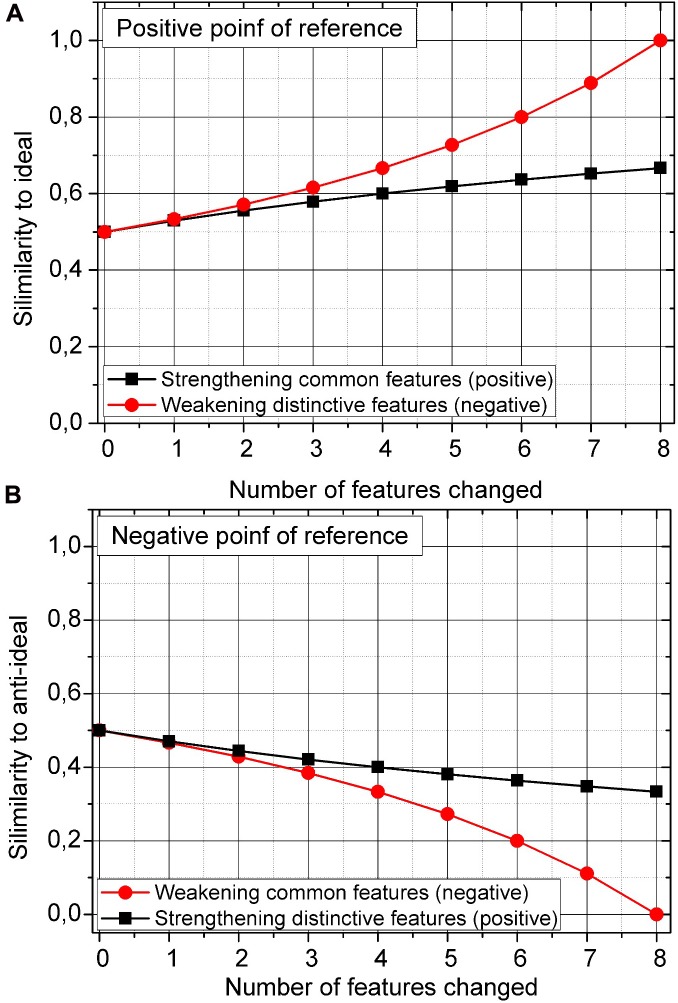
**(A)** Similarity (*S*) as a function of gradual strengthening or weakening of common and distinctive features for a positive point of reference (ideal). **(B)** Similarity (*S*) as a function of gradual strengthening or weakening of common and distinctive features for a negative point of reference (anti-ideal).

The ratio model can be expressed with the following equation:

$$s(a,b)=\frac{\left(I\cap R\right)}{\left(I\cap R\right)+\left(I-R\right)+\left(R-I\right)}$$

where the sum of common features that are shared by the real object and its ideal image is divided by the sum of all features, common (*I* ∩ *R*) and distinctive (*I* - *R*) + (*R* - *I*) belonging to both sets. Similarity is normalized so that *S* lies between 0 (no similarity) and 1 (full similarity). Depending on whether a feature is common or distinctive, its addition or removal will have a different effect on similarity as it is presented below in **Figures 2A,B**. **Figure 2** presents the changes in similarity between the real object and its ideal (**Figure 2A**) and its anti-ideal (**Figure 2B**) images which serve as reference points for comparisons.

Initially, the sets consisted of eight common and eight distinctive features, which resulted in *S* = 0.50 [8/(2 × 8) = 0.5]. The number of features was the same for both conditions (i.e., for ideal and anti-ideal images). First, we analyze the situation with a positive reference point (see **Figure 2A**). By gradually strengthening (or adding) common features (which value rose by 1, from 8 to 16), an increase in similarity to the ideal was relatively small. However, when the value of distinctive features was decreased by the same amount (from 8 to 0), the similarity to the positive reference point rose considerably. Although common and distinctive features were either increased or decreased by the same value (8), the operations resulted in different similarity values.

But a distinctive situation arises when the reference point is negative. As the result of a gradual decrease or removal of common features (from 8 to 0), similarity to the anti-ideal drops significantly. Comparatively, when the value of distinctive features is gradually increased by the same amount (from 8 to 16), the similarity to the anti-ideal rises only slightly.

The situations described show that operations in which common and distinctive features are strengthened/added or weakened/removed (as described in Tversky’s features of similarity) do not result in a linear function of similarity when used in open sets. This implies that judgments of similarity and dissimilarity are not complimentary. In this case, strengthening or weakening of distinctive features leads to two different similarity functions. This approach to similarity research extends the contrast model of similarity with the analysis of open sets.

Let us now consider the relationship between the reference point and the valence of common and distinctive features. When the ideal object serves as the reference point, distinctive features that only belong to the compared set are negative. However, when the anti-ideal constitutes the reference point, then negative features belong to the common features shared by both sets. As the analyzes showed, regardless of whether the ideal or anti-ideal served as the reference point, operations on negative features (their strengthening and weakening) had a stronger effect on changes in similarity than did the same operations conducted on positive features (see **Figures 2A,B**). The reduction of negative features pulls it closer to the ideal as well as distances it further away from the anti-ideal than the strengthening of positive features.

Hypotheses 1 and 2:

In our study, we empirically tested results of the conducted simulation based on the following hypotheses:

(1) Addition of negative (unique) features will distance the candidate further away from the ideal candidate than addition of positive (common) features will bring him or her closer to the ideal candidate.
(2) Addition of negative (common) features will bring the candidate closer to the bad candidate than addition of positive features will distance him or her away from the bad candidate.

## Materials and Methods

### Participants

Respondents were recruited from psychology students at a large university. All students volunteered for the study and were rewarded with extra credit for their participation. One hundred sixty-one participants took part in the experiment. The group was randomly divided into six research conditions. **Table 1** shows the number of respondents in each group and the outline of the study.

**Table 1:** Research conditions depending on the number of positive and negative features used in the description and similarity judgment.

	*N*	Number of positive features	Number of negative features	Type of operation
	**92**	**Similarity to an ideal politician**		

Group 1	35	5	5	Control group
Group 2	34	10	5	Addition of positive (common) features
Group 3	23	5	10	Addition of negative (unique) features

	**69**	**Similarity to a bad politician**		

Group 4	22	5	5	Control group
Group 5	23	10	5	Addition of positive (unique) features
Group 6	24	5	10	Addition of negative (common) features


The respondents were informed that they would participate in a study that aimed at measuring how positive and negative characteristics of political candidates affect attitude toward politicians. It was emphasized that the researchers were interested in each respondent’s answers and that the responses were anonymous. Gender, age, level of education, political beliefs (from 0 “extreme left” to 10 “extreme right”) and political engagement (from 0 “not at all interested in politics” to 10 “extremely interested in politics”) were controlled and they did not influence the results. On average, participants were moderately interested in politics (*M* = 4.34; *SD* = 2.579) and were neither extremely left- or right-wing oriented (*M* = 4.29; *SD* = 2.036).

### Procedure

In each of the conditions, respondents were presented with descriptions of a political candidate that, depending on the experimental group, consisted of a different number of positive and negative features (**Table 1**). The traits were selected based on their representativeness and drawn from previous research (detailed information within the “Materials” subsection below). Each participant read only one candidate profile. Depending on the group, respondents were asked to think about an “ideal politician” or a “bad politician” with the traits and abilities that he or she should possess in order to be representative for the particular category. The framing procedure was based on mental imagery instruction successfully used to promote thought listing (Sujan et al., 1993). In groups 1–3, respondents were asked to evaluate on an 11-point Likert scale a profiled politician’s similarity to an ideal politician and in groups 4–6 a profiled politician’s similarity to a bad politician. Lastly, participants were asked to evaluate their affect and preference toward the profiled candidate. The affective evaluation was measured with one item (“On the scale from 0 to 10, how much do you like this political candidate?” from 0 “strongly dislike” to 10 “strongly like”), along with their voting intention (“If the candidate would run for office, would you vote for him?” answered on an 11-point Likert scale, from 0 “very unlikely” to 10 “very likely”).

### Materials

In order to reliably measure the influence of positive and negative features on similarity judgments, affective evaluation and choice, we precisely controlled the traits that were used in the descriptions of political candidates. The MOCOM (Measure Of Consumption Object Meaning; Kleine and Kernan, 1988) method was adopted in order to determine which characteristics are most typical for the “ideal politician” and “bad politician” categories.

In the first stage, 96 students of three Polish universities (students of a technical university, psychology and applied linguistics departments of a graduate university) were asked to write down within 60 s all associations they had when they thought about an ideal politician (*N* = 49) and a bad politician (*N* = 47). Respondents were told that there were no right or wrong answers and that the researchers were interested only in each respondent’s opinions. It was emphasized that participants should write down all associations exactly in the same order as they came to mind. In total, participants retrieved 554 associations (258 for an ideal politician and 296 for a bad politician).

In the second stage, dominance scores for all associations were assigned according to Szalay and Deese’s recommendation (Szalay and Deese, 1978; Kleine and Kernan, 1988). The first response written by a respondent was given six points, the second five points, the third four points, the fourth to seventh responses three points, the eighth and ninth responses two points, and all subsequent responses were assigned 1 point. Next, all associations were analyzed and synonymous expressions were merged. All ambiguous characteristics were agreed upon based on the conclusions of three independent and competent judges (Cronbach, 1948). The inter-rater reliability was excellent, ICC (3, *k*) = 0.924, CI [0.866, 0.955] (Cicchetti, 1994). The analysis yielded 171 unique features for the “ideal politician” category and 194 unique features for the “bad politician” category. Three items from the “ideal politician” category and two from the “bad politician” category had to be removed as they referred to proper names of popular politicians and could not be generalized to other politicians. Finally, all dominance scores for common responses were summed across respondents. **Table 2** presents thirty features with highest values for both categories.

**Table 2:** Thirty most representative features for both categories together with their dominance scores.

Ideal politician		Bad politician	
**Feature**	**Sum of dominance scores**	**Feature**	**Sum of dominance scores**
Intelligent	54	Liar	63
Truthful	51	Corrupted	48
Honest	49	Incompetent	37
Just	31	Uneducated	26
Sincere	30	Stupid	21
Well-educated	20	Quarrelsome	20
Direct	16	Left-winger	20
Keeping promises	15	Radical	19
Lowering taxes	15	Intolerant	19
Right-winger	15	Dishonest	17
Good	13	Greedy	16
Open	13	Lazy	14
Committed	13	Thinking only about him/herself	13
Cares for wellbeing of others	12	Despotic	11
Emphatic	12	Not interested in the state	11
Competent	12	Not keeping election promises	11
Charismatic	11	Crook	11
Good speaker	11	Populist	11
Stable in his/her beliefs	11	Lacking culture	9
Loyal	10	Egoistic	9
Impartial	9	Hollow	9
Caring	9	Nepotistic	9
Altruistic	8	Freak	9
Trustworthy	8	Nut	8
Powerful	8	Disloyal	8
Ensuring security	7	Non-empathic	8
Eloquent	7	Arrogant	7
Consistent	7	Fraudulent	7
Active	6	Not interested in voters’ opinions	7
Liberal	6	Can’t talk well	7


The MOCOM (Kleine and Kernan, 1988) method has been effectively used in marketing and in a political context to measure brand associations (Cwalina and Falkowski, 2015). Derived features correspond well with research on features attributed to an ideal president and other political candidates in Poland’s 2000 presidential elections (Cwalina and Falkowski, 2000, 2006) and have been validated in other countries (Miller et al., 1986; Cwalina et al., 2005). Features such as competence, honesty, fairness, intelligence, education, activity and openness appear as universally accepted as important for a good political candidate. Although research on negative features in a candidate’s profile has been less abundant, there is some evidence for validity of unfavorable traits as well (Miller et al., 1986).

Derived features were used to construct candidates’ profiles which differed in the number of positive and negative traits. **Table 3** shows all features used in the descriptions. Each profile consisted of five positive and five negative features (the same for all conditions) and additional five traits (positive or negative dependent on the group). Characteristics with the highest values were selected, unless positive and negative traits were mutually exclusive. The mutual exclusion of features produced unequal scores for favorable and unfavorable characteristics. For that reason, less prominent characteristics were selected as additional positive and negative features. Bearing in mind that the study aimed to investigate potential positive–negative asymmetry, we made sure that the sum of dominance scores for both sets was similar so that any differences would result from the negativity effect and not the differences in particular traits that were used in the descriptions.

**Table 3:** Features used in the construction of candidates’ profiles and their dominance score.

Five positive features (across all conditions)		Five negative features (across all conditions)		Additional five positive features		Additional five negative features	
Intelligent	51	Incompetent	19	Open	13	Populist	11
Truthful	54	Uneducated	19	Committed	13	Stupid	21
Honest	49	Quarrelsome	20	Keeping promises	15	Despotic	11
Just	31	Radical	37	Cares for wellbeing of others	12	Greedy	16
Sincere	31	Intolerant	26	Empathic	12	Lacking culture	9
Total	215		121		65		68


## Results and Discussion

### Similarity to an Ideal Political Candidate and Similarity to a Bad Political Candidate

Hypothesis 1 was tested with an ANOVA model where similarity to the ideal candidate was the dependent variable. We analyzed the simple effect of the number of positive and negative features in the description of a political candidate. The control condition (five positive features, five negative features) was compared against the description that included more positive features (10 positive features, 5 negative features) and the one with more negative features (5 positive features and 10 negative features). The simple effect tested in the ANOVA model was significant, *F*(2, 88) = 4.239, *p* < 0.017, $\eta_{p}^{2}$ = 0.087. The *post hoc* analyzes (Sidak) revealed that the description of an unattractive political candidate (*M* = 3.391, *SD* = 2.349) differed significantly from the control condition (*M* = 5.00, *SD* = 2.326), *p* = 0.043. However, no significant difference between the description of an attractive political candidate (*M* = 5.147, *SD* = 2.524) and the control condition was observed, *p* = 0.992. Consistent with Hypothesis 1, the results showed that the negative effect of additional unfavorable features was greater than the positive effect of additional favorable features in judgments about similarity to the ideal candidate. The negative traits of the candidate distanced him or her further away from the image of an ideal politician than positive features pulled him or her toward it.

Hypothesis 2 predicted that additional unfavorable features of a candidate would make the candidate more similar to the image of a bad candidate than additional positive features would make him or her different from it. The hypothesis was tested with an ANOVA model where similarity to the bad candidate was the dependent variable. The simple effect of the number of positive and negative features in the description was insignificant, *F*(2, 66) = 2.266, *p* = 0.112, $\eta_{p}^{2}$ = 0.064, thus rejecting Hypothesis 2. The results of the analysis showed that neither the addition of positive features nor additional negative information had a significant impact on the similarity to a bad political candidate.

Here, we examined the effect of additional positive and negative features on judgments about the similarity to an ideal candidate and a bad candidate. It was hypothesized that negative information items would have a bigger effect on similarity ratings than operations on positive traits. The results partially confirmed our assumptions. When participants were asked to evaluate the similarity to an ideal candidate, unfavorable traits distanced the candidate further away from the image of a good politician than positive ones pulled him or her toward it. However, contrary to our expectations neither traits affected judgments on similarity to a bad political candidate, although the tendencies followed our hypotheses.

The greater effect of negative features on similarity to an ideal candidate was well explained by the negativity effect (Peeters and Czapinski, 1990; Baumeister et al., 2001) with a generally greater impact of negative stimuli in comparison to positive stimuli of the same absolute value. Yet the positive–negative asymmetry was not easily adapted to the second problem tested that questioned the similarity to a bad political candidate. It seemed that unfavorable information about the candidate carried more weight if he or she was compared to a better or ideal candidate but not if the opponent was perceived as worse.

Results were also sustained by research on the subject of optical illusions such as the Adelson Checker Shadow Illusion (Adelson, 1995) or the Müller-Lyer illusion (Otto-de Haart et al., 1999). Optical illusions are a popular visual phenomenon where an image is perceived in a manner that differs from objective reality. For instance, the Adelson Checker Shadow Illusion presented a checkerboard with light and dark squares that was partly shadowed by a cylinder and the Müller-Lyer phenomenon two lines that end in arrowheads. Despite the same objective features (the color of the squares and the length of the lines, respectively), the subjective perception of objects differed due to the context in which they were presented. Similarly, the judgment about two political candidates was moderated by the nature of the comparison. The negative traits stood out more in the politician’s profile if he or she was compared to an ideal political candidate than when juxtaposed with a bad politician.

The results are also in line with the findings on mental addition and subtraction of Dunning and Parpal (1989) who in a series of experiments showed that people perceived greater impact if they evaluated how an action would increase an outcome than when asked to determine the reduction in a result. Moreover, in one of the studies researchers observed a similar positive–negative asymmetry as the one present in our research. When they analyzed the differences in the effect of studying and not-studying on the number of test questions answered right or wrong, they noticed that although the asymmetry between addition and subtraction was present in both situations, the effect was mediated by the study situation. More precisely, the difference was visible only in the scenario when students considered the act of studying and was barely noticeable if the outcome was negative.

Our results bear resemblance to research on mental addition and subtraction. First, when a political candidate was juxtaposed with an image of an ideal politician, participants looked on how many more (or less) positive traits the candidate had compared to the point of reference. On the contrary, the comparison to a bad politician demanded that participants subtract any benefits that the candidate lacked. Furthermore, in all discussed studies, one object or an event was compared with its better or worse version and participants were asked to evaluate how these two things differed dependent on how a question was framed. In all of these cases, the differences were more prominent if the outcome was positive or in some way better. Although Dunning and Parpal (1989) looked into the hedonic value of an event as a possible mediator of the effect on mental addition and subtraction, they did not find justification for this claim.

### The Relationship between Similarity Judgments and Choice via Affect

It is our contention that affective value of the stimulus may be an important variable that explains less sensitivity to similarity judgments in situations with a negative point of reference. First, there is a wealth of experimental evidence demonstrating that affect influences how people think and make decisions. Emotions have been shown to interact with lower and higher level cognition that affects attention, memory, judgment risk assessment, and reasoning (Johnson and Tversky, 1983; Dolan, 2002; Blanchette and Richards, 2010; Yiend, 2010). Negative stimuli such as angry faces were noticed much faster than non-threatening or positive stimuli as observed by Öhman et al. (2001). Additionally, retrieval of positive autobiographical memories was shown to increase affect which in turn reduced information processing and made consumers more prone to persuasion that contained weak arguments (Baumgartner et al., 1992; Sujan et al., 1993). An important point in the discussion on the asymmetrical effect of positive and negative stimuli was also raised by Taylor (1991) who proposed the mobilization-minimization hypothesis. It postulated that a negative stimulus produced a strong and rapid response on the physiological, cognitive, and emotional level. This mobilization was followed by subsequent opposite processes that aimed at neutralization of the negative effect brought by the original stimulus. Accordingly, it was maintained that a powerful negative stimulus led to a final positive response as people were more motivated to avoid negative events than approach positive ones.

Ergo, we hypothesized that affect influenced willingness to vote for the candidate and mediated the relationship between the candidate’s evaluation (measured by similarity to an ideal candidate and similarity to a bad candidate) and voting intention. Furthermore, based on the findings of the first analysis, we hypothesized that due to increased affect evoked by thinking about negative stimuli, the effect would be stronger for negative comparisons.

Hypothesis 3:

Affect will mediate the relationship between similarity judgments (similarity to an ideal candidate and similarity to a bad candidate) and choice. The mediating effect will be stronger for the negative point of reference.

Verification of the mediating role of affect in the relationship between similarity judgments and choice was made using Process software (Hayes, 2013) and was conducted on standardized values (Preacher and Hayes, 2008; Cheung, 2009). The analyzes were run separately for the ideal and anti-ideal candidate but regardless of the condition, i.e., collapsing data from three different groups. The bias corrected bootstrap CI method was used in order to obtain optimal tests of the indirect effect. In order to evaluate the effect size of the mediating effect accurately both the *R*^2^_med_ (*R*-squared mediation effect size; Fairchild et al., 2009) and *K*^2^ (Kappa-squared; Preacher and Kelley, 2011) were calculated. **Table 4** shows descriptive statistics and correlations among the variables.

**Table 4:** Descriptive statistics and bivariate correlations for **(A)** affect, similarity to an ideal candidate and choice, **(B)** affect, similarity to a bad candidate and choice.

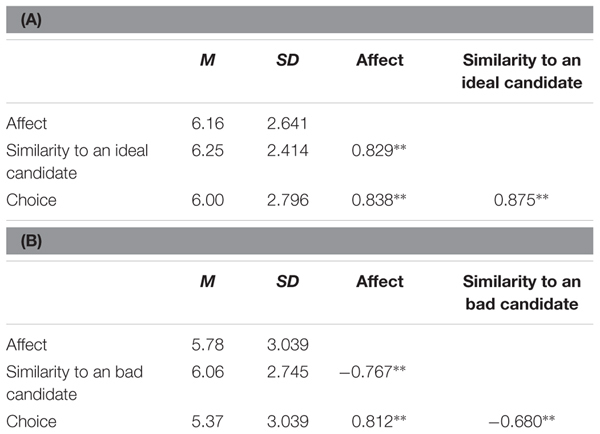

*^∗∗^*p* < 0.01.*

To test Hypothesis 3, we analyzed two models. In the first, we looked at the role that affect plays in the relationship between similarity to an ideal candidate and voting intention (choice). In the second, similarity to a bad political candidate was the independent variable.

**FIGURE 3:**
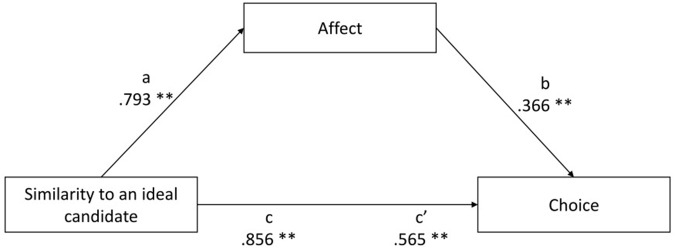
Model depicting the mediated effect similarity to an ideal political candidate via affect on choice.

In the first analysis, bootstrapping (*n* = 1000 bootstrap resamples, 95% confidence intervals) revealed a significant indirect effect of similarity to an ideal candidate on choice via affect (*a* × *b* = 0.290; *BootLLCI* = 0.143; *BootULCI* = 0.493), which supported Hypothesis 3. The direct effect remained statistically significant in the model (*c*′ = 0.565; *p* < 0.001). **Figure 3** shows the mediation influence of the similarity judgment on choice. The point estimate of *R*^2^_med_ was 0.662 (*BootLLCI* = 0.532; *BootULCI* = 0.754), that indicated that more than 66% of the variance in voting intention was attributable to the indirect effect of similarity to an ideal candidate through the rise of positive affect toward the candidate. The point estimate of *K*^2^ was 0.345; (*BootLLCI* = 0.155; *BootULCI* = 0.512). According to the standards, both statistics suggest a large effect size of the indirect effect (Cohen, 1992; Preacher and Kelley, 2011).

**FIGURE 4:**
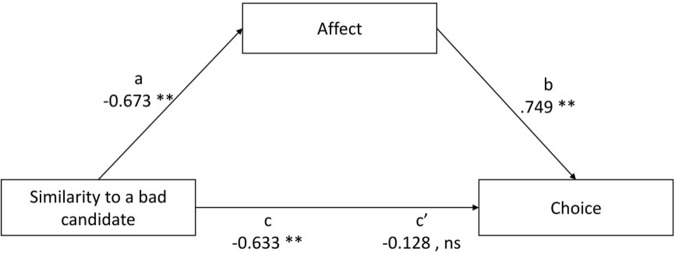
Model depicting the mediated effect similarity to a bad political candidate via affect on choice.

Next, the second model was tested. The analysis of the mediating role of affect in the relationship between the similarity to a bad political candidate and choice revealed a significant indirect effect (*a* × *b* = 0.504; *BootLLCI* = -0.726; *BootULCI* = -0.343), while the direct effect became statistically insignificant (*c*′ = -0.128; *p* = 0.218). The results supported Hypothesis 3. **Figure 4** shows the mediation influence of the similarity judgment on choice. The point estimate of *R*^2^_med_ was 0.454 (*BootLLCI* = 0.245; *BootULCI* = 0.631). The point estimate of K^2^ was 0.483; (*BootLLCI* = 0.350; *BootULCI* = 0.616). Based on the guidelines for the effect size interpretation, both statistics indicate a large effect size of the mediating effect.

Finally, to test whether the positive or negative point of reference made the mediating effect of affect stronger, we compared the two models. Both *R* squared mediation effect size and Kappa squared indicated large effect sizes of the indirect effects in two models. Although both statistics are recognized as good measures of the effect size, Kappa squared has been recommended as a more reliable parameter (Preacher and Kelley, 2011). The comparison of Kappa squared for these two models showed a bigger proportion of the indirect effect if similarity to a bad political candidate was the independent variable. Furthermore, in the second model, the direct effect *c*′ becomes insignificant which, according to the causal steps approach suggested by Baron and Kenny (1986), is a sign of “perfect mediation” (p. 1177). The insignificant *c*′ was proposed as a method to judge the effect size of an indirect effect (Baron and Kenny, 1986; Preacher and Kelley, 2011; cf. MacKinnon et al., 2002). Based on Kappa squared and the insignificant direct effect in the model with similarity to a bad political candidate, we conclude that affect played a more prominent role in the relationship between a negative point of reference and choice than when people compared the candidate to an ideal political candidate. Based on these findings, it may be argued that negative features activate stronger emotions than positive characteristics. Although the strength of emotions was not directly measured, the correlations between the affective evaluation and similarity judgments seem to justify such an assumption. The correlations were conducted for three groups separately (1) control condition, (2) positive features added, (3) negative features added and showed a stronger relationship when negative features were added. For comparisons to an ideal candidate the following correlations were found: for the control condition, *r* = 0.789, *p* < 0.001, for additional positive features added, *r* = 0.872, *p* < 0.001, for additional negative features added, *r* = 0.887, *p* < 0.001; for comparisons to an anti-ideal candidate, *r* = -0.703, *p* < 0.001, for additional positive features added, *r* = -0.704, *p* < 0.001, for additional negative features added, *r* = -0.882, *p* < 0.001. The results of mediational analyzes and correlations between similarity judgments and affect yet again seem to prove that after all “bad is stronger than good” (Baumeister et al., 2001).

## General Discussion

The purpose of the conducted research was to analyze the mechanics of the negativity effect which has been an object of numerous psychological studies, both on the cognitive and emotional level. Based on previous research (Falkowski et al., 2018) and a simulation study, we used Tversky’s (1977) research on features of similarity to analyze the relationship between common and distinctive features in open sets. To date, the original model has been most often studied with reference to closed sets and artificial stimuli such as schematic faces or letters that do not fully capture the complexities of natural objects in physical and social environments. In our study, we used the extended model to open sets and analyzed how an addition or removal of character features affected the evaluation of a more complex object like a political candidate. What’s more, although Falkowski et al. (2018) proposed a theoretical analysis of the asymmetrical effect of operations on common and distinctive features in brand evaluation, the model was not empirically tested. Our study provides an empirical verification and validation of the hypothesized effects.

The use of the model of features of similarity on open sets gave better understanding of the differences in the effect of positive and negative features on similarity judgments and preference formation. In our study, we focused on two issues: the valence of features and similarity judgments. We analyzed how additional favorable and unfavorable information about a political candidate affects his or her image and influences judgments about his or her similarity to an ideal politician and a bad politician. The results showed that additional unfavorable traits had a stronger negative effect on similarity to an ideal candidate than positive features. Once again our findings gave support to the negativity effect by showing that negative characteristics of a political candidate tarnished his or her image more than positive traits made it more attractive.

Our research has shown that the positive–negative asymmetry can be interpreted not only on the emotional level but also in reference to cognitive processes. Literature provides much evidence of the stronger effect of negative information on preference formation that results in more thorough information processing (Fiske and Taylor, 1991), faster reaction times (Pratto and John, 1991), and stronger avoidance behavior (Wentura et al., 2000). Additionally, studies on similarity judgments showed that the nature of comparison affected preference and led to different choices (Dhar et al., 2000). Our study significantly adds to the research on the interplay between cognition and emotion with regard to the negativity effect.

First, we demonstrated and provided an understanding of the ways in which features’ valence can affect similarity judgments and choice. Our study showed that the stronger effect of negative features was visible only in situations where a political candidate was compared to an ideal politician but disappeared when comparisons were made to a bad politician. This is consistent with earlier findings (e.g., Dunning and Parpal, 1989) and points to a moderating effect of the reference point in similarity judgments.

Next, in order to explain this effect, we tested the mediating role of affect in the relationship between similarity judgments and choice. As hypothesized, the analyses revealed that affective evaluation of the candidate mediated the relationship between similarity judgments and voting intention. Also, the comparison of these two models showed that the mediating effect was stronger for a negative reference point. This finding gave grounds to the assumption that negative comparisons, leading to the activation of negative features, create stronger emotions than positive features of comparable strength. This effect has been well documented in the literature on positive–negative asymmetry (see Peeters and Czapinski, 1990; Baumeister et al., 2001).

The application of the contrast model of similarity to the study of positive–negative asymmetry was used to explain numerous instances of the negativity effect. One of such phenomena, often presented in consumer and political marketing, is comparative advertising where one brand points to the inferior performance of an otherwise similar competitive brand or when it tries to use the other brand as a negative point of reference as it presents itself. This view was studied by Bizer et al. (2011) who tested how framing one’s choice (i.e., supporting one’s candidate vs. being against an opposing candidate) affected voting intention. The researchers showed that opposition attitudes (the result of negative valence framing) were stronger and more resistant to persuasion than supporting attitudes but they did not explain the underlying mechanism of valence framing. Based on simulations described in the introductory section as well as the empirical results of our study, it may be assumed that operations on negative features are stronger than operations on positive characteristics of a comparable strength. Furthermore, the results of mediational analyzes suggested that negative valence framing activated stronger negative emotions that mediate the relationship between comparison judgments and voting intention.

Finally, the research showed that the positive–negative asymmetry was not universal but was dependent on the reference point. Our results indicate that the stronger effect of negative features was present only in situations where the candidate was compared to an ideal political candidate but disappeared when he or she was juxtaposed to a bad politician. Typically, when choosing from a variety of options, it is natural that one wants to select the best option and hence assumes a positive reference point. As shown in our paper and numerous other studies, in these situations negative attributes tend to have a stronger effect and lead to avoidance behavior. Yet, it may be possible that the effect does not hold for the negative reference point. More precisely, in situations in which all options are suboptimal (for example, all candidates are bad), even a relatively weak positive feature may have a strong effect on the candidate’s evaluation. In this instance, the positivity effect may be argued. Still, the effect may depend not only on the reference point but also on the initial similarity between two compared options. If two sets are initially very different, then changes in common features appear as more effective. However, if two options are very similar, then the operations on distinctive characteristics will likely have a stronger effect. The adoption of the contrast model of similarity to open sets together with the distinction of positive and negative reference points allows precision in predicting the asymmetrical effect of addition and deletion of common and distinctive features to sets which differ in their initial similarity. The empirical validation of these effects constitutes an interesting research gap that should be addressed in further studies.

### Limitations and Further Studies

Although our study added to the understanding of the positive–negative asymmetry in political decision making and provided more insight into the mediational effect of affective evaluation in the relationship between similarity judgments and voting intention, it is important to consider its limitations. Even though we managed to point to the affect as a mediator between similarity judgments and choice, we only partially explained the complex nature of this relationship. Future studies should aim to investigate in more detail cognitive processes that are involved in this relationship and further mediate the path between affective evaluation and choice. Moreover, the simulations described in the introductory section hold true for the initial similarity coefficient around 0.5 but the relationship between common and distinctive features is different for very low and very high values of similarity (as visible on the diagrams). Further studies should test to what an extent experimental results are in accordance with the theoretical model for different values of initial similarity. Furthermore, we realize that the present experiment concentrated only on the evaluation of political candidates. Yet, we believe that the effects observed in our research are more general in nature and can be extended to other fields. As a final point, the same effect may be measured using different methods with regard to the way that positive and negative features are manipulated. In the present study, we tested how adding and removing of certain characteristics affect similarity judgments, affective evaluation and decision making but in further studies we plan to manipulate the strength of particular features (e.g., downplaying or magnifying their importance).

## Ethics Statement

This study was carried out in accordance with the recommendations of Ethics Committee for Scientific Research at Psychology Department of SWPS University of Social Sciences and Humanities, Warsaw with written informed consent from all subjects. All subjects gave written informed consent in accordance with the Declaration of Helsinki. The protocol was approved by the Ethics Committee for Scientific Research at Psychology Department of SWPS University of Social Sciences and Humanities, Warsaw.

## Author Contributions

AF substantial contribution to the conception of the paper and experimental design; the interpretation of data for the paper; drafting and revising the paper; Final approval of the version to be published; agreement to be accountable for all aspects of the work in ensuring that questions related to the accuracy or integrity of any part of the work are appropriately investigated and resolved. MJ substantial contribution to the conception of the paper and experimental design; the acquisition, analysis and interpretation of data for the paper; the interpretation of data for the paper; drafting the paper; Agreement to be accountable for all aspects of the work in ensuring that questions related to the accuracy or integrity of any part of the work are appropriately investigated and resolved.

## Conflict of Interest Statement

The authors declare that the research was conducted in the absence of any commercial or financial relationships that could be construed as a potential conflict of interest.
